# Hyperthermic intraperitoneal chemotherapy (HIPEC) in combined treatment of locally advanced and intraperitonealy disseminated gastric cancer: A retrospective cooperative Central‐Eastern European study

**DOI:** 10.1002/cam4.2204

**Published:** 2019-04-29

**Authors:** Roman Yarema, Jerzy Mielko, Taras Fetsych, Myron Ohorchak, Magdalena Skorzewska, Karol Rawicz‐Pruszyński, Artem Mashukov, Viatcheslav Maksimovsky, Tomasz Jastrzębski, Wojciech Polkowski, Petro Gyrya, Yuriy Kovalchuk, Victor Safiyan, Ivan Karelin, Viatcheslav Kopetskiy, Olena Kolesnik, Yuriy Kondratskiy, Marius Paskonis

**Affiliations:** ^1^ Danylo Halytsky Lviv National Medical University Lviv Ukraine; ^2^ Medical University of Lublin Lublin Poland; ^3^ Lviv State Oncological Regional Treatment and Diagnostic Center Lviv Ukraine; ^4^ Odessa National Medical University Odessa Ukraine; ^5^ Medical University of Gdańsk Gdańsk Poland; ^6^ National Cancer Institute Kyiv Ukraine; ^7^ Vilnius universtiy hospital Santaros klinikos Vilnius Lithuania

**Keywords:** cytoreductive surgery, gastric cancer, hyperthermic intraperitoneal chemotherapy, peritoneal metastases

## Abstract

**Background and Objectives:**

Clinical experience in Western Europe suggests that cytoreductive surgery (CRS) and hyperthermic intraperitoneal chemotherapy (HIPEC) are promising methods in the management of gastric cancer (GC) with peritoneal metastases. However, there are almost no data on such treatment results in patient from Central‐Eastern European population.

**Methods:**

A retrospective cooperative study was performed at 6 Central‐Eastern European HIPEC centers. HIPEC was used in 117 patients for the following indications: treatment of GC with limited overt peritoneal metastases (n = 70), adjuvant setting after radical gastrectomy (n = 37) and palliative approach for elimination of severe ascites without gastrectomy (n = 10).

**Results:**

Postoperative morbidity and mortality rates were 29.1% and 5.1%, respectively. Median overall survival in the groups with therapeutic, adjuvant, and palliative indications was 12.6, 34, and 3.5 months. The only long‐term survivors occurred in the group with peritoneal cancer index (PCI) of 0‐6 points without survival difference in groups with PCI 7‐12 vs PCI 13 or more points.

**Conclusions:**

GC patients with limited peritoneal metastases can benefit from CRS + HIPEC. Hyperthermic intraperitoneal chemotherapy could be an effective method of adjuvant treatment of GC with a high risk of intraperitoneal progression. No long‐term survival may be expected after palliative approach to HIPEC.

## 1 INTRODUCTION

Despite the reduction in incidence rate, gastric cancer (GC) remains one of the most frequent oncological diseases with almost 1 milion new cases per year globally.^1^ Gastric cancer is the sixth most common cancer and the fourth cause of cancer‐related deaths in Europe.^2^

The most frequent pattern of GC metastasis is peritoneal carcinomatosis,^3^ which is diagnosed in 14%‐30% of all GC patients.^4^, ^5^ Peritoneal recurrence after radical surgery develops in 34%‐60% of patients and remains the main cause of GC patient death.^6^, ^7^

Palliative chemotherapy remains the standard of care for such patients, but ensures the median overall survival (OS) only at the level of 8 months without 5‐year survival.^5^, ^8^ The use of targeted therapy is in most cases restricted to intestinal type GC, characterized by hematogenous cancer spread.^9^

During the last 2 decades, the paradigm of treatment of intraperitoneally disseminated tumors has been changing by way of introduction of cytoreductive surgery (CRS) and hyperthermic intraperitoneal chemotherapy (HIPEC). Such approach to treatment has already proved its efficiency in peritoneal carcinomatosis from colorectal cancer, peritoneal pseudomyxoma, and mesothelioma.^10^, ^11^ However, the clinical experience of using CRS + HIPEC in GC patients remains limited.^12^

By now optimistic results of one randomized^13^ and a few retrospective clinical studies of CRS + HIPEC effectiveness in GC patients with implantation metastasis coming from East^14^, ^15^ and Western Europe^16^ have been published. Also, 2 meta‐analyses of the studies of effectiveness of adjuvant intraperitoneal chemotherapy in patients with locally‐advanced GC (mainly studies from Eastern clinics) have been published.^17^, ^18^ However, there are almost no data on such treatment results in the population of GC patients from Central‐Eastern Europe.

The aim of the study was to create a clinical registry of HIPEC for patients with GC from Central and Eastern Europe, as well as to analyse short‐ and long‐term outcomes.

## 2 MATERIAL AND METHODS

With a view to studying the effectiveness of using HIPEC in combined treatment of locally advanced and intraperitonealy disseminated GC, invitations to participate in the retrospective cooperative study were sent to 13 Central‐Eastern European HIPEC centers that have had experience with GC patients. Data were collected from 6 centers that responded: 3 centers in Ukraine (Lviv, Odesa, Kyiv), 2 centers in Poland (Lublin, Gdansk), and 1 center in Lithuania (Vilnius).

### 2.1 Patients and specimens

Hyperthermic intraperitoneal chemotherapy effectiveness in the combined treatment of 117 patients with intraperitonealy disseminated or locally advanced GC with high risk of intraperitoneal progression was analyzed. The patients were on inpatient treatment in the 6 aforesaid Central‐Eastern European HIPEC centers between 2008 and 2017 (retrospective cooperative clinical study). Fifty‐six males and 61 females participated in the study. The mean age was 54.1 ± 10.9 years (range, 22‐75 years). The age of the patients ranged from 22 to 75 years old, the average age was 54.1 ± 10.9 years old. The diagnosis of GC in all patients was verified morphologically prior to the treatment offset. The GC stage was evaluated based on criteria of the TNM 7th edition classification (2009). Written informed consent was obtained from all patients.

HIPEC was performed in 117 GC patients for the following indications:

- Curative group: treatment of GC with limited overt peritoneal metastases (n = 70).
- Adjuvant group: adjuvant/proactive setting after potentially radical gastrectomy performed in patients with locally advanced GC and high risk of intraperitoneal progression (n = 37).

- Palliative group: palliative approach for elimination of severe ascites without gastrectomy (n = 10).

Main clinical and pathological characteristics of patients are presented in Table 1.

**Table 1:** Clinical and pathological characteristics of 117 GC patients

Characteristics		number, n (%)
Sex	Male	56 (48)
	Female	61 (52)
Primary gastric cancer location	Antral part	16 (13.7)
	Corpus	44 (37.6)
	Proximal part	1 (0.9)
	Antral part + corpus	26 (22.2)
	Corpus + proximal part	2 (1.7)
	Subtotal or total lesion	24 (20.5)
	Unknown	4 (3.4)
Tumour histology	G2	8 (6.8)
	G3	25 (21.4)
	G4	48 (41)
	Signet ring cell	24 (20.5)
	Mucinous	5 (4.3)
	Other	1 (0.9)
	Unknown	6 (5.1)
Curative group (n = 70)		
Stage of peritoneal carcinomatosis according to Japanese classification (JGCA)	P0 (CY1)	4 (5.7)
	P1	28 (40)
	P2	31 (44.3)
	P3	7 (10)
Peritoneal cancer index, points	0‐6	49 (70)
	7‐12	13 (18.6)
	13 and more	7 (10)
	Unknown	1 (1.4)
Mean peritoneal cancer index, points	5,6 ± 3,6 (0‐19)	
Ascites	Present	3 (4.3)
	Absent	67 (95.7)
pT (TNM 7, 2009)	pT4a	54 (77.2)
	pT4b	15 (21.4)
	Unknown	1 (1.4)
pN	pN0	15 (21.4)
	pN+	38 (54.3)
	Unknown	17 (24.3)
Completeness of cytoreduction score	CC‐0	50 (71.4)
	CC‐1	15 (21.4)
	CC‐2,3	5 (7.2)
Postoperative systemic chemotherapy	Yes	44 (62.9)
	No	17 (24.3)
	Unknown	9 (12.8)
Adjuvant group (n = 37)		
pT (TNM 7, 2009)	pT4a	29 (78.4)
	pT4b	8 (21.6)
Total circular tumor infiltration of stomach serous membrane	Present	10 (27)
	Absent	27 (73)
pN	pN0	12 (32.4)
	pN+	24 (64.9)
	Unknown	1 (2.7)
Stage (TNM 7, 2009)	IIB	10 (27)
	IIIA	2 (5.4)
	IIIB	12 (32.4)
	IIIC	13 (35.2)
Postoperative systemic chemotherapy	Yes	8 (21.6)
	No	28 (75.7)
	Unknown	1 (2.7)
Palliative group (n = 10)		
Mean volume of ascitic fluid, litres	5,5 ± 1,4 (3,5‐8)	
Mean peritoneal cancer index, points	30,6 ± 6,1 (15‐39)	

### 2.2 Curative group

Twenty‐one (30%) patients of the group were given neoadjuvant chemotherapy.

In the Curative group combined treatment included CRS (total or subtotal gastrectomy, regional lymph node dissection and various peritonectomy procedures according to P. Sugarbaker) in combination with HIPEC followed by systemic chemotherapy.

The mean surgical peritoneal cancer index (PCI) score, which was defined intraoperatively, was 5.6 ± 3.6 (range 0‐19) points. The completeness of cytoreduction (CC) score was evaluated after the surgery: CC‐0 cytoreduction was performed in 50 (71.4%) patients, CC‐1—in 15 (21.4%), and CC‐2 and 3—in 5 (7.2%).

In 29 (41.4%) patients in the given group D0 and D1 lymph node dissection was performed and in 41 (58.6%) patients D1+ and D2 lymph node dissection was performed. The presence of lymphogenous metastases in removed regional lymph nodes were histologically confirmed in 38 (54.3%) patients.

After CRS and HIPEC 44 (62.9%) patients of the Curative group received a systemic chemotherapy. Chemotherapy regimens were as follows: CF was used in 13 (29.6%) patients, EOX—in 7 (15.9%), XELOX—in 6 (13.7%), CAF—in 4 (9.1%), ECF—in 3 (6.8%), Tegafur—in 3 (6.8%), FLO—in 2 (4.5%), FOLFIRI—in 2 (4.5%), and other—in 4 (9.1%).

### 2.3 Adjuvant group

In the Adjuvant group combined treatment included potentially radical surgery (total or subtotal gastrectomy with regional lymph node dissection) in combination with HIPEC in adjuvant regime followed by systemic chemotherapy.

In 13 (35.1%) patients of this group D0 and D1 lymph node dissection was carried out, in 24 (64.9%) patients D1+ and D2 lymph node dissection was carried out. The presence of lymphogenous metastases in removed regional lymph nodes were histologically confirmed in 24 (64.9%) patients.

After surgery and HIPEC 8 (21.6%) patients from the Adjuvant group received a systemic chemotherapy. Chemotherapy regimens were as follows: XELOX was used in 3 (37.5%) patients, EOX—in 2 (25%), CAF—in 2 (25%), and CF—in 1 (12.5%).

### 2.4 Palliative group

In the Palliative group combined treatment was applied using palliative surgery (laparotomy, ascites evacuation) in combination with HIPEC for management of recurrent ascites.

The average number of laparocentesis procedures before combined treatment of patients of the given group was 1.1 ± 0.8 (range 0‐3). The mean volume of peritoneal fluid retrieved from the abdominal cavity intraoperatively amounted to 5.5 ± 1.4 liters (range 3.5‐8 liters). Mean surgical PCI was 30.6 ± 6.1 (range 15‐39) points.

### 2.5 HIPEC procedure

The HIPEC procedures were performed by the “closed” technique in the majority of cases—in 110 (94%) patients, while the “Coliseum” (open) technique was used in 7 (6%) cases. The duration of HIPEC was 90 minutes in 80 (68.4%) cases, 60 minutes in 14 (12%) and 30 minutes in 23 (19.6%). Mean intraabdominal temperature was 42.7 ± 0.78°C (range 40‐44). For conducting HIPEC the following chemotherapeutic agents were applied intraperitoneally: Mitomycin 12.5 mg/m^2^ + Cisplatine 75 mg/m^2^ was used in 61 (52.1%) patients, Oxaliplatine 460 mg/m^2^—in 23 (19.7%) patients, Mitomycin 10‐15 mg/m^2^ or 10 mg/l—in 23 (19.7%) patients, Cisplatine 75 mg/m^2^ + Doxorubicin 15 mg/m^2^—in 7 (6%) patients and Cisplatine 75 mg/m^2^—in 3 (2.5%). Thirty‐one (26.5%) trial patients were given bidirectional chemotherapy (HIPEC plus intraoperative intravenous 5‐FU infusion).

### 2.6 Follow‐up

Patients were regularly followed up after the surgery. Abdominal ultrasound was performed every 3 months and chest X‐ray every 6 months during the first 2 postoperative years and every 6 months thereafter. Abdominal computerized tomography was performed every 6‐12 months. The disease‐free survival (DFS) was measured from the date of the surgery to the date of recurrence, metastasis occurrence, death or last follow‐up. The OS was measured from the date of the surgery to the date of death or last follow‐up.

### 2.7 Statistical analysis

Statistical analysis of primary data was performed with SPSS 22 and Statistica 6 software. Censored Kaplan‐Meier method was used to study the cumulative survival of patients, whereas the reliability of the survival level difference in certain groups was determined using a log‐rank coefficient. The multivariate analysis was performed using the Wald test.

## 3 RESULTS

### 3.1 Immediate results

Postoperative morbidity rate after surgery with HIPEC was 29.1% (31 patients). Twenty (17.1%) patients developed surgical complications. Anastomotic leak was observed in 7 (6.5%) patients, abdominal collections without gastrointestinal leak in 5 patients, postoperative pancreatitis—4, intraabdominal bleeding—2, bowel obstruction—1, mesenteric thrombosis—1, and small bowel perforation in 1 patient. Seventeen (14.5%) patients developed surgical complications, which required relaparotomy. Eight (6.8%) patients suffered from HIPEC‐related complications grade III‐IV: leukopenia grade III‐IV was noted in 3 patients, nephrotoxicity grade III‐IV—2, intestinal paresis—2 and pyrexia during HIPEC—1. Nine (7.7%) patients developed general complications: pneumonia—5 patients, pleural effusion—3, and heart complications—1 patient. The 30‐day postoperative mortality rate was 5.1% (6 patients).

### 3.2 Long‐term outcomes

Median follow‐up was 48.7 months for patients after a combined treatment.

**Figure 1:**
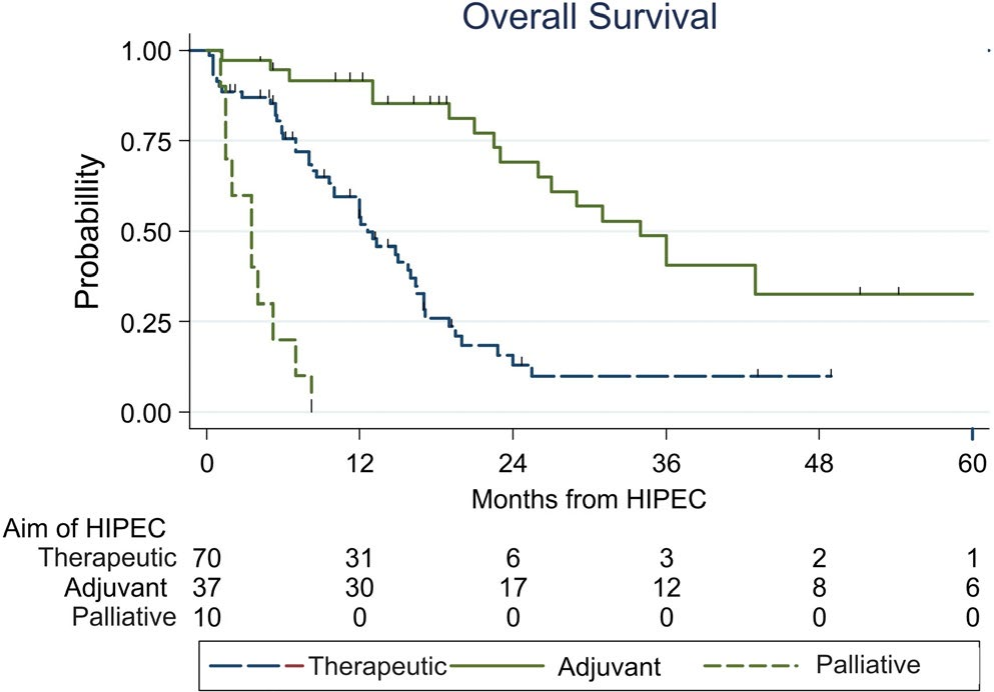
The (OS) rate of 117 (GC) patients who underwent combined treatment with (HIPEC). GC, gastric cancer; HIPEC, hyperthermic intraperitoneal chemotherapy; OS, overall survival

The DFS and OS rates of 117 GC patients differed statistically depending on the aim of performing HIPEC (Table 2, Figure 1).

**Table 2:** The DFS and OS rate of 117 GC patients who underwent combined treatment with HIPEC

Aim of HIPEC	Patients (n)	Disease‐free survival			Overall survival		
		1 year	Median (months)	P logrank	1 year	Median (months)	P logrank
Curative group	70	41.8%	10.0	<0.0001	53.8%	12.6	<0.0001
Adjuvant group	37	82.3%	28.0		91.7%	34.0	
Palliative group	10	0%	2.5		0%	3.5	

Abbreviations: GC, gastric cancer; HIPEC, hyperthermic intraperitoneal chemotherapy.

Out of 106 uncensored patients (11 patients were censored because of death as a result of surgical complications or intercurrent pathology, or dropped out of the follow‐up) the progression of the disease developed in 66 (62.3%). In the Curative group, out of 60 uncensored patients, disease progression developed in 47 (78.3%). The most frequent cause of that was intraperitoneal relapse, which occured in 39 (65%) patients. In the Adjuvant group, out of 36 uncensored patients, disease progression developed in 9 (25%) patients, in all cases due to intraperitoneal relapse. All of the 10 patients of the Palliative group had a rapid intraperitoneal progression.

### 3.3 Curative group

Univariate analysis identified the following prognostic factors of OS in the Curative group (Table 3): rate of PCI, the presence of ascites, and depth of tumor invasion (pT4a vs pT4b). The following factors were found on the threshold of statistical confidence: peritoneal carcinomatosis stage according to Japanese GC Association (JGCA) and CC score. For a statistically significant effect of DFS, the following were important: histological structure, stage of peritoneal carcinomatosis (JGCA), PCI score, presence of ascites, depth of tumor invasion and CC score. Out of these, in the multivariate analysis only depth of tumor invasion retained statistical significance for OS (HR = 2.32; 95% CI: 1.1‐4.88, *P* = 0.026). Stage of peritoneal carcinomatosis (JGCA) (HR = 2.01; 95% CI: 0.95‐4.28, *P* = 0.053) and CC score (HR = 3.3; 95% CI: 1.35‐8.03, *P* = 0.009) retained statistical significance for DFS.

**Table 3:** Univariate analysis of prognostic factors in GC patients from Curative group with CRS/HIPEC

Variable	Patients (*n* = 70)	1 year OS	Median OS (months)	*P* *(logrank)*	1 year DFS	Median DFS (months)	*P* *(logrank)*
Gender				0.086			0.16
Male	28	59.1%	19.0		48.2%	12.0	
Female	42	50.5%	12.1		36.9%	10.0	
Age				0.80			0.81
<40	7	20.0%	10.0		30.0%	8.0	
40‐49	12	61.1%	15.8		37.5%	8.0	
50‐59	29	57.7%	12.6		34.0%	10.0	
≥60	22	53.1%	16.0		57.8%	13.0	
Histological structure				0.32			**0.008**
G2	6	33.3%	8.6		0%	5.0	
G3	9	55.6%	22.8		66.7%	18.0	
G4	30	60.1%	14.8		48.6%	12.0	
Signet‐ring cell	15	43.7%	10.0		25.0%	6.0	
Mucinous	3	100%	12.0		100%	‐‐‐	
Other	1	100%	12.0				
Peritoneal stage (JGCA)				0.084			**0.0001**
P1	28	65.2%	16.0		69.0%	16.0	
P2	31	46.3%	12.0		31.5%	9.0	
P3	7	28.6%	7.0		0%	4.0	
P0(Cyt+)	4	50.0%	10.0		25.0%	6.0	
PCI, points				**0.034**			**0.0002**
0‐6	49	64.1%	15.0		54.7%	13.0	
≥ 7	20	47.3%	8.2		8.9%	4.0	
pN				0.82			0.96
pN0	15	65.0%	13.0		50.0%	10.0	
pN+	38	58.2%	14.8		41.4%	11.0	
pT (TNM 7, 2009)				**0.0009**			**0.001**
pT4a	54	67.9%	14.8		48.1%	12.0	
pT4b	15	33.9%	7.0		18.5%	4.0	
Ascites				**0.034**			**0.0015**
Present	3	33.3%	2.8		0%	1.5	
Absent	67	61.1%	13.0		43.4%	11.0	
Lymph node dissection				0.82			0.33
D0, D1	29	54.2%	12.0		23.0%	8.0	
D1+, D2	41	62.5%	15.0		52.4%	12.5	
Cytoreduction score				0.093			**0.0001**
CC‐0	50	64.0%	15.0		55.9%	13.0	
CC‐1 −2 −3	20	43.7%	8.6		0	4.0	
Systemic chemotherapy				0.27			0.49
Yes	44	69.1%	15.0		40.2%	10.0	
No	17	47.5%	9.6		38.9%	8.0	

Abbreviations: CRS, cytoreductive surgery; DFS, disease‐free survival; GC, gastric cancer; HIPEC, hyperthermic intraperitoneal chemotherapy; JGCA, Japanese Gastric Cancer Association; OS, overall survival; PCI, peritoneal cancer index.

Bold type indicated statistically significant indications of P (logrank).

**Figure 2:**
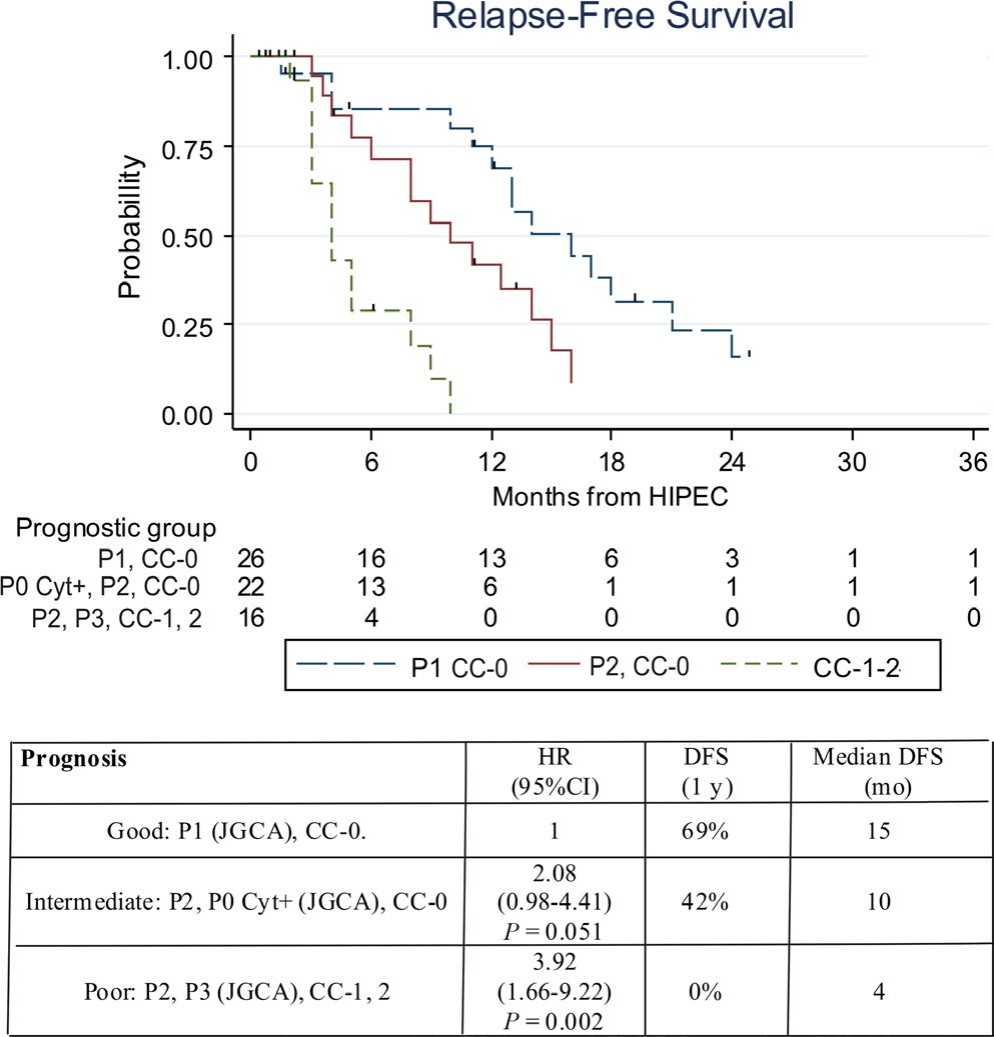
Prognostic score for (DFS) of (GC) patients with peritoneal metastases who underwent combined therapy with (HIPEC). DFS, disease‐free survival; overall survival; GC, gastric cancer; HIPEC, hyperthermic intraperitoneal chemotherapy

Multivariate analysis results made it possible to construct a prognostic score for DFS for Curative group of patients with good, intermediate, and poor prognosis (Figure 2).

At applying this prognostic score to OS, the results are not as discriminatory with median OS 16, 12.6, and 8.6 months in the three prognostic groups, respectively (*P* = 0.21).

### 3.4 Influence of PCI on the prognosis

**Figure 3:**
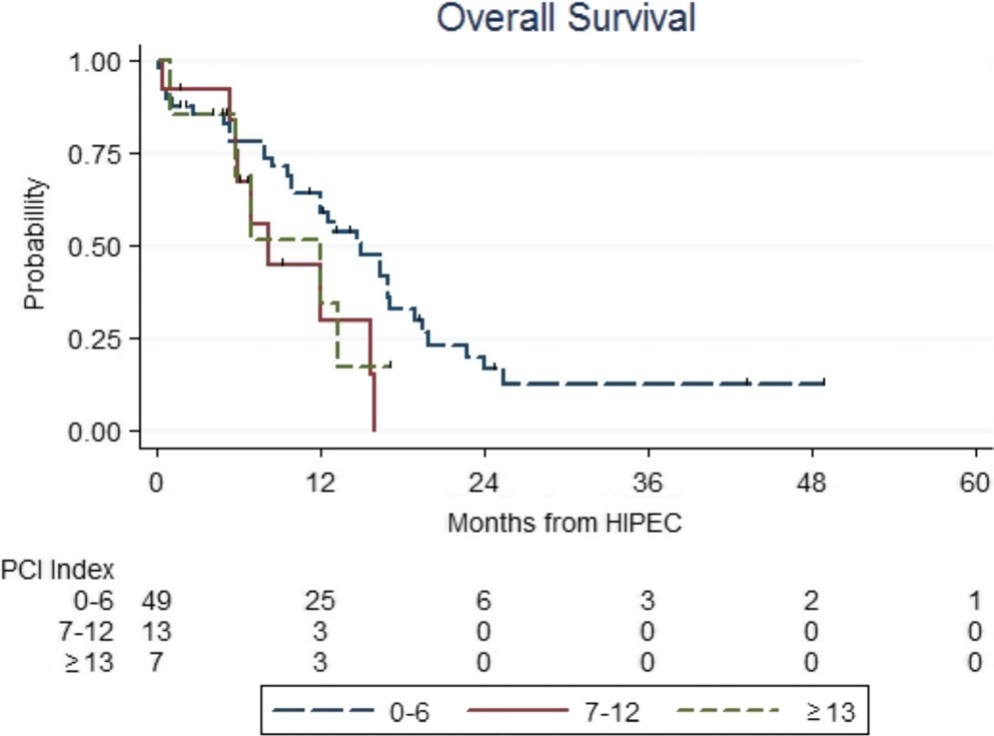
Prognosis of (GC) patients with peritoneal metastases who underwent combined therapy with (HIPEC) according to (PCI) rate. GC, gastric cancer; HIPEC, hyperthermic intraperitoneal chemotherapy; PCI, peritoneal cancer index

At the division of patients of Curative group into 3 categories according to PCI level, no statistically significant difference in survival rates was found between the group of patients with the PCI 7‐12 points and those with PCI 13 or more points (Figure 3). The only long‐term survivors occurred in the group of patients with PCI of 0‐6 points—median OS reached 15 months, whereas in patients with PCI ≥ 7 points the median OS reached 8.2 months (HR = 2.02; 95% CI: 1.03‐3.93, *P* = 0.04).

### 3.5 Adjuvant group

In the adjuvant setting, only one variable in the univariate analysis was significantly related to OS and DFS and that was tumor histology. Notably patients with undifferentiated (G4) GC had a significantly worse prognosis with a HR = 3.21 (95% CI: 1.22‐8.44, *P* = 0.0162) for OS and a HR = 5.00 (95% CI: 1.84‐13.58, *P* = 0.0009) for DFS. On the threshold of statistical confidence, the index of regional lymph node status was found (pN) (HR = 2.48; 95% CI: 0.82‐7.59, *P* = 0.085).

In the multivariate analysis undifferentiated (G4) histology retained statistical significance for OS (HR = 2.85; 95% CI: 1.07‐7.56, *P* = 0.036) and for DFS (HR = 4.28; 95% CI: 1.55‐11.82, *P* = 0.005).

### 3.6 Palliative group

In 10 patients of the Palliative group, HIPEC was performed as part of combined treatment with the aim of recurrent ascites elimination, median OS reached only 3.5 months and DFS—only 2.5 months. In this group only 2 (20%) patients had to undergo repetitive laparocentesis procedure due to ascites recurrence.

## 4 DISCUSSION

After the first application of HIPEC in GC patients in 1988 by the Japanese surgeon S. Fujimoto,^19^ this direction has been researched intensively. However, up till now the effectiveness of such treatment of GC patients remains somewhat unclear, and the issue of the necessity and methods of its application remains disputable and requires further research.^20^, ^21^

To date, results of 2 major retrospective studies concerning this problem have been published in literature. Thus, in 2005 Y. Yonemura et al from Shizuoka (Japan)^14^ analyzed group of 107 patients with intraperitoneally disseminated GC treated with the use of HIPEC after conducting aggressive CRS. The authors reported a median OS of 11.5 months, but also for the first time in many years 5‐year survival rate (6.7%) was estimated for a group of patients with such unfavorable prognosis. In patients with complete cytoreduction (CC‐0) the median OS and 5‐year survival rate were higher—15.5 months and 27%, respectively.

In 2010 O. Glehen et al from Lyon (France)^16^ published results of a French retrospective multi‐center study based on the analysis of treatment outcomes of 159 patients from 15 centers. The median OS of 9.2 months was reached as well as 1‐, 3‐, and 5‐year survival of 43%, 18%, and 13%, respectively. For patients with complete cytoreduction (CC‐0) the median OS was 15.0 months and 1‐, 3‐, and 5‐year survival reached 61%, 30%, and 23%, respectively. The 3‐year survival rate was not reported for patients with PCI exceeding 12 points, as well as 6‐month survival rate when PCI exceeded 19 points. Therefore, the authors concluded on the effectiveness of such combined treatment only in patients with limited and resectable peritoneal carcinomatosis (with a PCI score less than 12 points).

The necessity to achieve complete cytoreduction is also emphasized in a number of publications.^12^, ^22^ In 2008 a consensus decision was made to accept PCI below 12 points as threshold for performing CRS + HIPEC in GC patients.^23^ F. Coccolini et al in a recent metaanalysis^12^ confirmed the PCI score threshold below 12 points.

Long‐term results of this cooperative study in the Curative group were similar to the results from the French study: median OS and 1‐year survival rate in the general group are reported at the level 12.6 months and 53.8% respectively, in the complete cytoreduction group (CC‐0)—15 months and 64% respectively. Median OS in patients with CC‐0 is almost twice as high as the survival of patients with CC‐1, 2, 3 (15 vs 8.6 months), but the difference in survival was found on the threshold of statistical confidence.

However, we have received some new unexpected results. Namely, no significant difference is observed between the survival outcomes of patients with PCI 7‐12 points and those with PCI ≥ 13. Long‐time survival was only achieved in patients with PCI 0‐6 points: median OS reached 15 months compared with 8.2 months in patients with PCI > 6. Similar unexpected results were also reported in a recent study by C. Caro et al,^24^ which prepares the ground for further discussion and possible revision of recommendations in the future.

In our study the stage of peritoneal carcinomatosis (JGCA) was another independent prognostic factor for DFS besides complete cytoreduction, which enabled suggesting the construction of a prognostic score for DFS. Most patients from the Curative group had intraperitoneal relapse (65%) after CRS + HIPEC. However, in 8 (13.3%) patients the disease progressed at the absence of implantation metastasis, which suggests the effect of such treatment on intraperitoneal carcinogenesis processes. The answer to the question of CRS + HIPEC effectiveness in such patients can be given by the results of the German phase III trial GASTRIPEC that are awaited in the near future.

Locally advanced GC in a vast number of cases is accompanied by subclinical peritoneal dissemination at the time of operation, which is confirmed by peritoneal carcinomatosis manifestation during the first few months or years after treatment. Obviously, such patients require combined treatment methods. Positive results of using HIPEC in the adjuvant mode in GC patients are confirmed by 2 meta‐analyses.^17^, ^18^ In the Adjuvant group of this study the median OS and 1‐year survival rates at the level of 34 months and 91.7%, respectively, were achieved at a quite low level of intraperitoneal relapse—25%. It is expected that the European randomized study GASTRICHIP will confirm the effectiveness of HIPEC in adjuvant mode in GC patients.

The literature has solitary reports on the palliative way of using HIPEC for elimination of severe ascites in patients with diffuse peritoneal carcinomatosis.^25^ The results in the Palliative group of this study confirmed ascites elimination in all patients, however, with no effect on survival.

## 5 CONCLUSIONS

Central‐Eastern European population of GC patients with limited peritoneal metastases can benefit from CRS + HIPEC. The only long‐term survivors occurred in the group with PCI 0‐6 points without survival difference between groups with PCI 7‐12 and PCI 13 or more points. HIPEC could be an effective method of adjuvant treatment of GC with a high risk of intraperitoneal progression. Aside from ascites elimination, no long‐term survival may be expected after palliative approach to HIPEC.

## DISCLOSURES AND FUNDING SOURCES

The authors declare no proprietary, financial, or other personal interests related to this article.

## SYNOPSIS FOR TABLE OF CONTENTS

This study shows that gastric cancer patients with limited peritoneal metastases can benefit from CRS and HIPEC. HIPEC could be an effective method of adjuvant treatment of gastric cancer with a high risk of intraperitoneal progression.
